# Interface coupling-induced enhancement of magnetoimpedance effect in heterogeneous nanobrush by adjusting textures of Co nanowires

**DOI:** 10.1186/1556-276X-8-471

**Published:** 2013-11-09

**Authors:** Yi Zhang, Juan Dong, Xiaojun Sun, Qingfang Liu, Jianbo Wang

**Affiliations:** 1 Key Laboratory for Magnetism and Magnetic Materials of the Ministry of Education, Institute of Applied Magnetics, Lanzhou University, Lanzhou 730000, People's Republic of China

**Keywords:** Nanobrush, Magnetoimpedance, Nanowire texture, Micromagnetic simulation

## Abstract

Interface coupling-induced and interface coupling-enhanced magnetoimpedance (MI) effect in heterogeneous nanobrush has been investigated. The nanobrush is composed of Fe_25_Ni_75_ nanofilm and textured hexagonal close-packed cobalt nanowire array, respectively fabricated by RF magnetron sputtering and electrochemical deposition. The design of this structure is based on the vortex distribution of magnetic moments in thin film, which can be induced by the exchange coupling effect at the interfaces of the nanobrush. The texture of nanowires plays an important role in the MI effect of the nanobrush, which is regulated by controlling the pH values and temperatures of the deposition process. The ‘parallel’ and ‘perpendicular’ coupling models were used to explain the different MI results of the nanobrush with cobalt nanowires, which have (100) and (002) textures, respectively. The optimized MI effect of the nanobrush brought by (100) nanowires can be magnified by 300% with more than 80%/Oe magnetic sensitivity at a low frequency, which has great application potentials in low-frequency MI sensors.

## Background

In recent years, low-dimensional nanomaterials have attracted considerable attention due to their potential application in many areas [1]. One-dimensional nanowires with large shape anisotropy and surface area have attracted much attention, which will be useful in a wealth of applications that include catalysis, magnetic recording, and some physical fundamental researches [2,3]. Two-dimensional magnetic nanofilm is widely used for various kinds of magnetic sensors, planar inductors, and so on [4,5]. Great efforts have been made to combine different structures for three-dimensional multifunction materials. For instance, Qin et al. fabricated a microfiber-nanowire hybrid structure for energy scavenging, and Yan et al*.* fabricated three-dimensional metal-graphene nanotube multifunctional hybrid materials [6,7]. As a typical hybrid nanostructure, nanobrush has been under extensive studies as one of the nanodevices for its special characters [8,9]. In a magnetic composite material, the exchange coupling effect at the interface is significant [10,11]. In order to investigate its influence on nanobrush, a heterogeneous nanobrush with magnetic film and different textured cobalt nanowires is dwelt on in detail in this paper. Different coupling models at the interface induced by different cobalt crystal textures have been investigated. The structure shows great performance as far as the magnetoimpedance effect is concerned.

The magnetoimpedance (MI) effect has been considered as a potential physical effect with higher field sensitivity and better signal intensity for magnetic sensors than the giant magnetoresistance effect [12]. Since MI changes with the external direct current (dc) magnetic field or applied dc/alternating current (ac) current, it is possible to design MI sensors used to measure magnetic fields or dc/ac currents. Several kinds of industrial and engineering applications of MI sensors have been proposed and realized to date, such as in the field of traffic controls, automobile uses, and biomedical sensors [13-16]. Amorphous wires, ribbons, and composited soft magnetic wires are traditional MI materials [12,17,18]. Normally, the diameter of amorphous wires and the thickness of ribbons are up to micrometer scale. With the rapid development of nanomaterials, the size of magnetic sensors is projected to reach nanoscale. The traditional MI materials cannot satisfy the desired size, and multilayer film MI materials have increasingly become the hot spot. However, the multilayer films may come into being only when an obvious MI ratio reaches gigahertz [19,20], and it is not good for the application of MI sensors. Therefore, finding new kinds of nanomaterials, which can have both an obvious MI effect and a rapid magnetic response at low frequency, is a great challenge.

The MI effect is normally attributed to a combination of skin effect and high sensitivity of transverse permeability to the external applied field. In a magnetic medium, the skin depth is dependent on the transverse magnetic permeability (*μ*_t_) through $\delta_{m}=c/\sqrt{2\mathrm{\pi f}\mu_{t}\sigma }$_,_ where *σ* and *μ*_t_, respectively, are the electrical conductivity and the transverse permeability of the ferromagnetic material. For amorphous ribbons and wires, many ways have been tried to improve the MI ratio, which include annealing, ion irradiation, glass coating, and patterning [21-23]. Essentially, all the above approaches to enhance the MI ratio are based on the changes of magnetic domain and induced transverse distribution of magnetic moments [12]. For films, the sandwich structure is an effective approach to depress the skin effect and improve the MI ratio, but a low MI ratio and high working frequency pose major negative factors for applications. Obviously, it is urgent to solve the problem of how to induce transverse moment distribution and enhance the MI ratio in the nanomaterial.

The structure of heterogeneous nanobrush with strong interface coupling may provide new ideas for these challenges. As our former works turn out, the giant MI (GMI) ratio has been enlarged than the single FeNi film on an anodized aluminum oxide (AAO) template, and the exchange coupling effect between nanowires and film has been supposed to be the main reason of the enhanced MI ratio [24]. However, how the exchange coupling effect acting on MI results is unclear. In this paper, a kind of magnetic nanobrush, which combines Fe_75_Ni_25_ film and cobalt nanowire arrays with different textures, is prepared. The obvious diversity of MI curves has been apparently observed in (100)- and (002)-textured nanobrushes. Micromagnetic simulation is used to analyze the phenomenon.

## Methods

**Figure 1:**
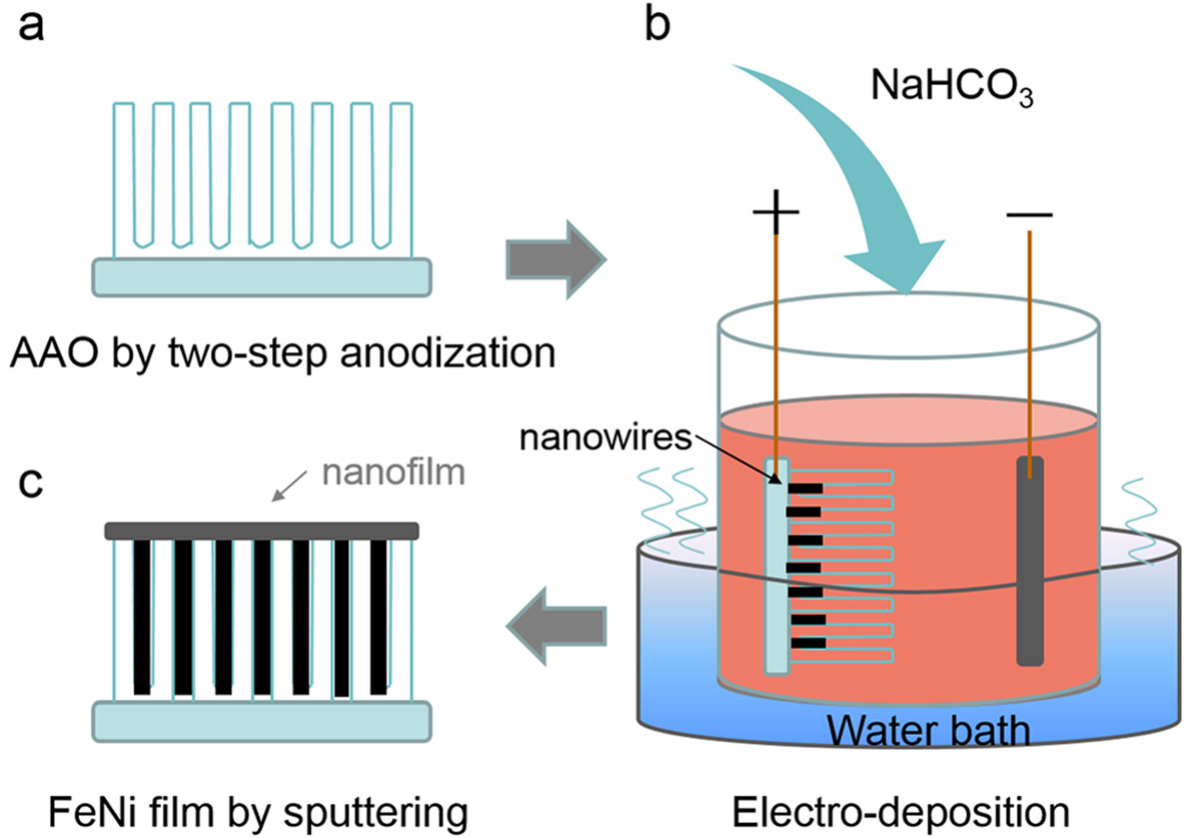
**Preparation of the heterogeneous nanobrush with different textures. (a)** A regular AAO template was achieved via two-step oxidation, **(b)** electrochemical deposition textured cobalt nanowires by regulating pH values and proper water bath, and **(c)** FeNi film covered the surface by magnetron sputtering.

Figure 1 shows the preparation of the heterogeneous nanobrush with different textures based on AAO templates and magnetron sputtering. Self-ordered anodic aluminum oxide templates were prepared by a two-step anodization process [25]. As shown in Figure 1a, the 20- and 50-nm AAO templates were prepared by two-step anodization in sulfuric acid and oxalic acid solutions, respectively. The Co nanowires were deposited by alternating current electrodeposition. The formation of textures is very sensitive to the pH value and temperature. The saturated NaHCO_3_ solution was added dropwise to regulate the pH value, and the water bath was used to control the deposition temperature (Figure 1b). For the 50-nm AAO templates, the (100) texture was deposited when pH = 6.2 and the water bath was 60°C, and the (100), (002), and (101) mixed textures were deposited when pH = 4.5 and the water bath was 20°C. For the 20-nm templates, (100), (002), and (100) and (002) mixed textures were deposited under 40°C, pH = 4.5; 20°C, pH = 6.4; and 10°C, pH = 6.4, respectively. Once collected, a 100-nm-thick Fe_25_Ni_75_ film was sputtered on the surface of AAO templates with a common base pressure below 3 × 10^-5^ Pa and a processing Ar pressure of 0.4 Pa (Figure 1c). The RF power was 140 W, and the duration of deposition was 30 min. Moreover, the FeNi film would have to cover the top of the AAO template, and the surface of the sample was conductive.

X-ray diffraction (XRD) confirmed the composition of the nanowire arrays. The surface topography and nanostructure were observed via scanning electron microscopy (SEM). The magneto-optic Kerr effect (MOKE) was used to obtain the surface magnetic properties of the composite material. Micromagnetic simulations were performed with the three-dimensional (3D) object-oriented micromagnetic framework (OOMMF) method [8]. The exchange constants of the film and wires, respectively, were 1.3 × 10^-11^ and 1.75 × 10^-11^ J/m. The damping parameter *α* was 0.5, the mesh size was 5 × 5 × 5 nm^3^, and the saturation magnetization of the permalloy film and Co nanowires, respectively, were 8.6 × 10^5^ and 1.42 × 10^6^ A/m. Prior to MI measurement, the samples were tailored into small pieces with a length of 20 mm and width of 3 mm. An impedance analyzer (Agilent 4294A, Agilent Technologies, Inc., Santa Clara, CA, USA) was used in the four-terminal contact mode to measure the impedance (*Z*). The magnitude of the driving voltage is 500 mV. All the electronic instruments were controlled using LabVIEW (National Instruments, Austin, TX, USA).

## Results and discussion

**Figure 2:**
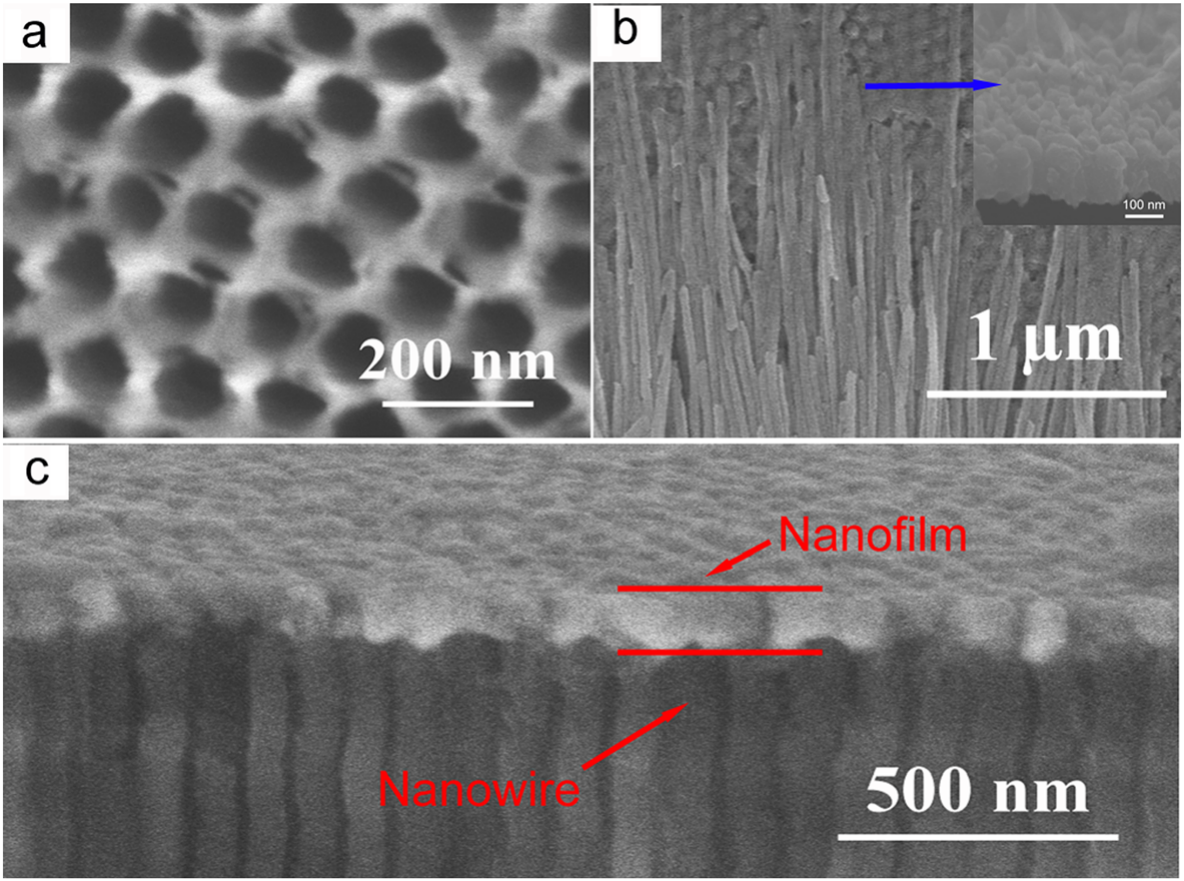
**Surface topography of AAO templates and the cross section of the nanobrush. (a)** AAO templates with diameters of 50 nm, **(b)** interface of the nanobrush after the AAO framework was removed, and **(c)** profile of the nanobrush with 50-nm nanowire array.

The AAO templates were used to fabricate the nanobrush, and the cross profile of the nanobrush was revealed from the microscopic investigations. A scanning electron microscopy image of self-ordered AAO templates is taken in top view (Figure 2a). The uniform SEM contrast observed from the side (Figure 2b) proves the homogeneous Co deposition inside the nanowires of the whole AAO templates and along their whole length. Figure 2c shows the interface of the nanobrush after the AAO framework was removed via NaOH bath. It can be seen clearly from the inset that nanowires and nanofilm connect tightly.

**Figure 3:**
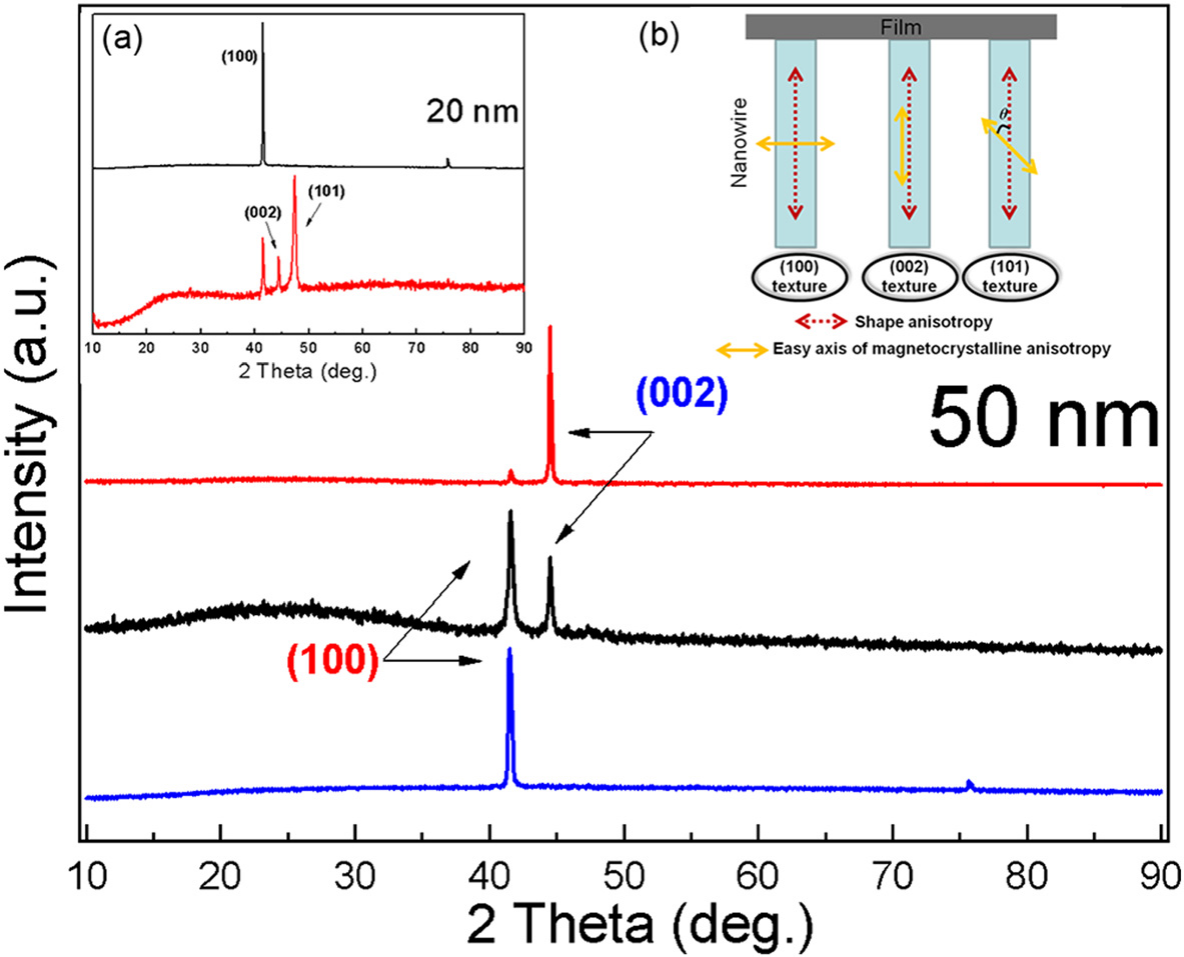
**XRD patterns of 50-nm nanowires with (100), (002), and (100) and (002) mixed textures. (a)** 20-nm nanowires with (100) and (100), (002), and (101) mixed texture and **(b)** schematic diagrams of the competition between the shape anisotropy and the magnetocrystalline anisotropy.

The enhanced MI performance of nanobrush depends on the exchange coupling effect of the interface between nanowires and films. Although the ac current flows through the top FeNi film, the crystal texture of cobalt nanowires strongly influences the exchange coupling effect at the interface. As we know, the magnetocrystalline anisotropy constant *K*_1_ of bulk hexagonal close-packed (hcp) cobalt is 5 × 10^6^ erg/cm^3^ at room temperature, which is the largest value among the d-band ferromagnetic metals such as Fe, Co, and Ni, and it nearly balances the shape anisotropy (*K*_s_ = 6 × 10^6^ erg/cm^3^) of magnetic nanowire [26]. Thus, purposefully controlling the crystal texture of cobalt nanowires is considered to be valuable for investigating the MI properties at the film part of the nanobrush due to the exchange coupling effect at the interface [24]. Figure 3 shows XRD patterns of the cobalt nanowire arrays with different textures, and the inset shows the schematic diagrams of the competition between the shape anisotropy and the magnetocrystalline anisotropy. The (100) texture means the easy axis of magnetocrystalline anisotropy is perpendicular to the long axis of nanowires. In other words, the magnetic moments of nanowires at the interface are parallel to the FeNi film [27,28]. The (002) texture means the easy axis of magnetocrystalline anisotropy is parallel to the long axis of nanowires (Figure 3b). For the 20-nm samples, the position of the peak center is 41.680°, which is consistent with the standard diffraction of hcp Co (100) (41.683°). The (101) and (002) peaks appear when the pH value of the electrolyte reaches 4.5 under room temperature. For the 50-nm samples, the (002) peak (44.264°) was prepared at the pH value of 6.4 and temperature of 20°C.

**Figure 4:**
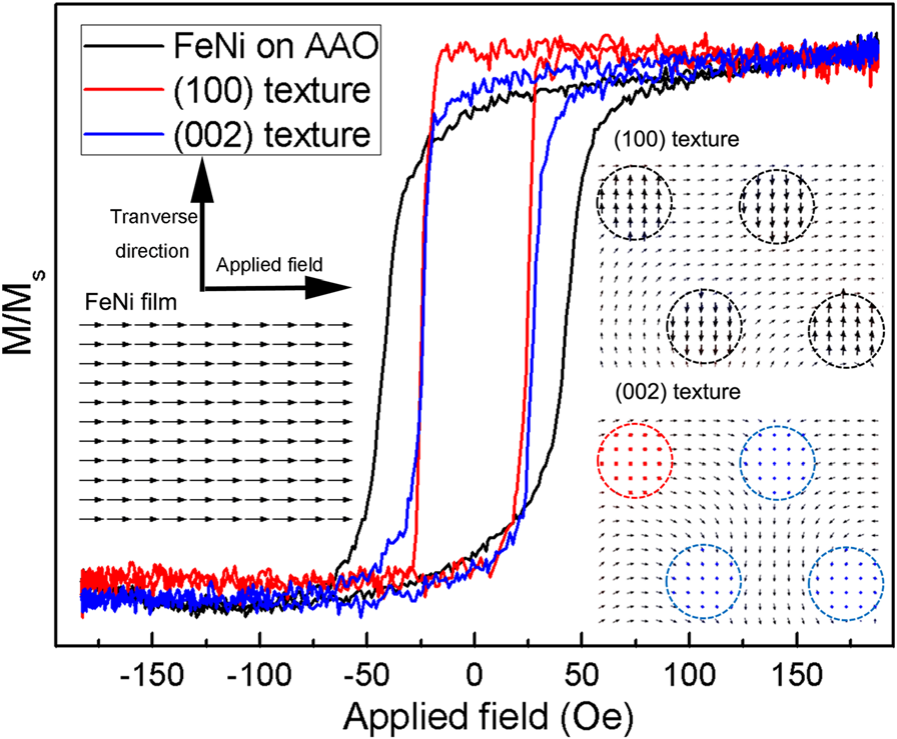
**Static magnetic properties of nanobrushes with different textures.** Micromagnetic simulations of the top surface magnetic properties of the nanobrush are shown in the inset.

Static magnetic properties of the top films of the nanobrush are shown in Figure 4. The (100)-textured sample shows the smallest coercivity and a good aspect ratio. For the FeNi film deposited on AAO templates, surface defects may destroy the soft magnetic properties. The magnetic moment distribution induced by the interface coupling effect conveys different characteristics, which may result in different performances of magnetoimpedance effect of the nanobrush. The insets of Figure 4 show the distribution of magnetic moments of the top film in the nanobrush. The nanobrush combined with permalloy film and hcp Co nanowires is used during simulation. The thickness of the permalloy film and the diameter of Co nanowires are both 50 nm. An external field applied in the plane of the film is 50 Oe. The direction of magnetic moments is denoted by the arrows. As shown in the inset, the magnetic moments of a single film lie in the plane. When an external field was applied, the magnetic moments turn to the field direction. Transverse moments can hardly be found. However, for the films of the nanobrush, a strong exchange coupling effect takes place at the interface of the nanofilm and nanowire array, leading to a vortex distribution of magnetic moment, and lot moments turn to be perpendicular to the applied field. Thus, the MI effect may be intensified due to the transverse component magnetic moments. For the (100) texture, magnetic moments distribute perpendicular to the long axis of nanowires. At the interface, planar vortex distribution of film moments is induced by the exchange coupling effect. Most transverse magnetic moments will enhance the transverse permeability when an external field is applied. By contrast, the magnetic moments in (002) texture nanowires are along the long axis, and the induced vortex distributions will be perpendicular to the film plane. Although many transverse moments have been observed, the perpendicular moments may block the increase of transverse moments and reduce the transverse permeability.

**Figure 5:**
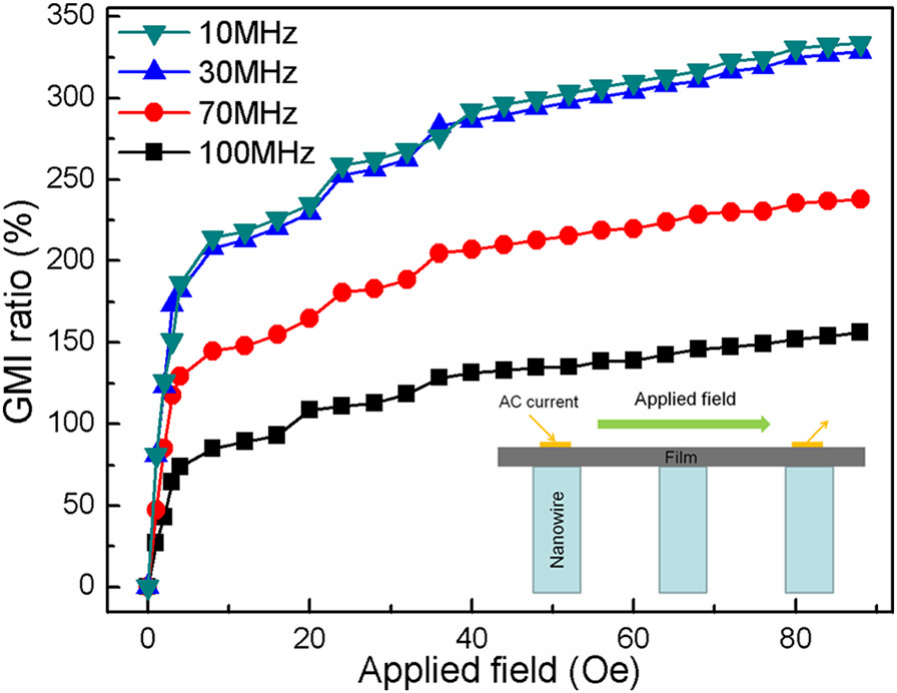
MI ratio of nanobrush at different current frequencies when applied field is 0 to 86 Oe.

Figure 5 shows the MI ratio under different applied fields of the nanobrush in combination with the FeNi film and 20-nm (100)-textured cobalt nanowires at different frequencies (*f* = 10, 30, 70, and 100 MHz). As the inset shows, the applied field is along the direction of the ac current, which is parallel to the FeNi film. On the one hand, with the externally applied magnetic field increasing, the MI ratio increases sharply and an obvious change of the MI ratio takes place in small fields. The MI curves can be explained by the magnetization rotation model [29], in which the transverse magnetic permeability plays an important role. On the other hand, four different frequencies were marked at the measurement of field dependence GMI properties. It is found that the optimal GMI result is at 10 MHz, as a consequence of the contribution of the permeability from both domain wall motion and magnetization rotation. With the increase in frequency, reduction in GMI is related to the domain walls becoming strongly damped by eddy currents and only magnetization rotation contributes to GMI [12,30].

**Figure 6:**
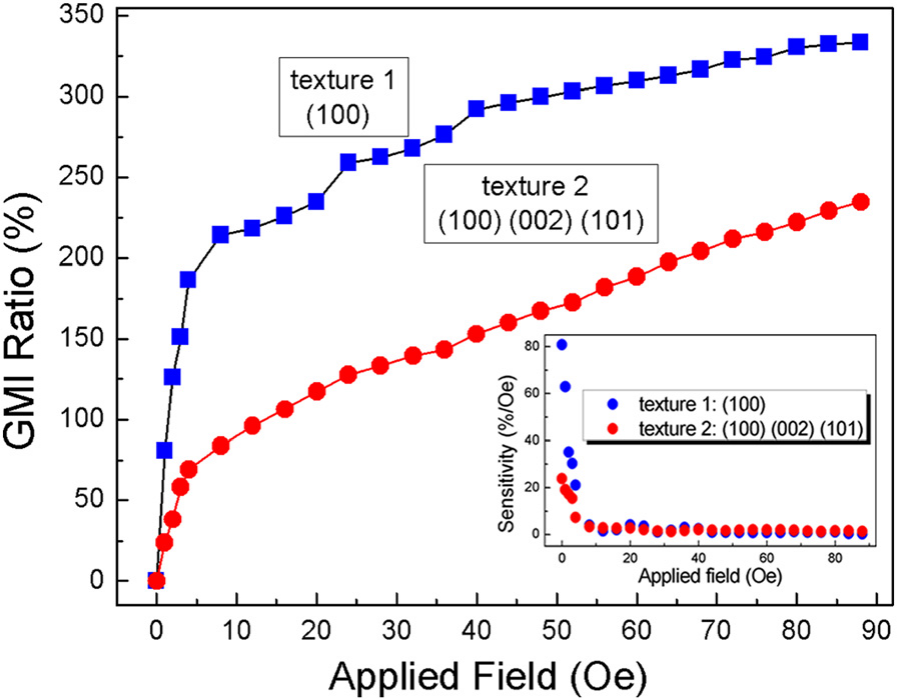
MI ratio and magnetic response of the nanobrush with 20-nm textured nanowires.

Figure 6 shows the field dependence of the magnetoimpedance effect of the nanobrush in combination with the FeNi film and 20-nm textured cobalt nanowires at a frequency of 10 MHz. The (100)-textured nanobrush shows a better MI ratio, which reaches up to more than 300%. The result is better than our former work [24]. The MI ratio of the mixed textured ((100), (101), and (002)) nanobrush is about 200%. The MI ratio with applied magnetic field is expressed as Δ*Z*/*Z* = [*Z*(*H*_ex_) - *Z*(*H*_0_)]/*Z*(*H*_0_) × 100%, where *Z*(*H*_ex_) and *Z*(*H*_0_) represent the impedance with and without a magnetic field H, respectively. Considering the exchange coupling effect, the MI curves in the nanobrush appear to be different from the traditional materials. The MI ratio will not drop dramatically until the external applied field is up to the saturation field [24]. The (100) texture contributes to the magnetic moments of the interface to distribute on the film; on the contrary, the appearance of the (002) texture may assist the moment to be perpendicular to the film. If the magnetic moments are parallel to the film, the permeability will be enhanced than the situation that the moments are perpendicular to the film. So the MI ratio of the (100) texture is much better than that of the (002) texture.

It should be emphasized that not only the MI ratio but also the magnetic response is important for high-performance sensor application. The inset of Figure 6 shows the magnetic response to the different textures of 20-nm nanowires. The sensitivity (*S*) of the MI is defined as follows: *S* (%/Oe) = (Δ*Z*/*Z*)/Δ*H*, where Δ*H* is the change of the magnetic field. At a very small external applied field, the field sensitivities of the MI effect of the 20-nm nanobrush are 80% and 25%. Afterwards, it begins to decrease and approach a value which is approximately equal to zero. The MI ratio and sensitivity of the nanobrush with FeNi film and 20-nm (100)-textured Co nanowires are higher than some typical MI results of single film and multilayer film [31,32].

**Figure 7:**
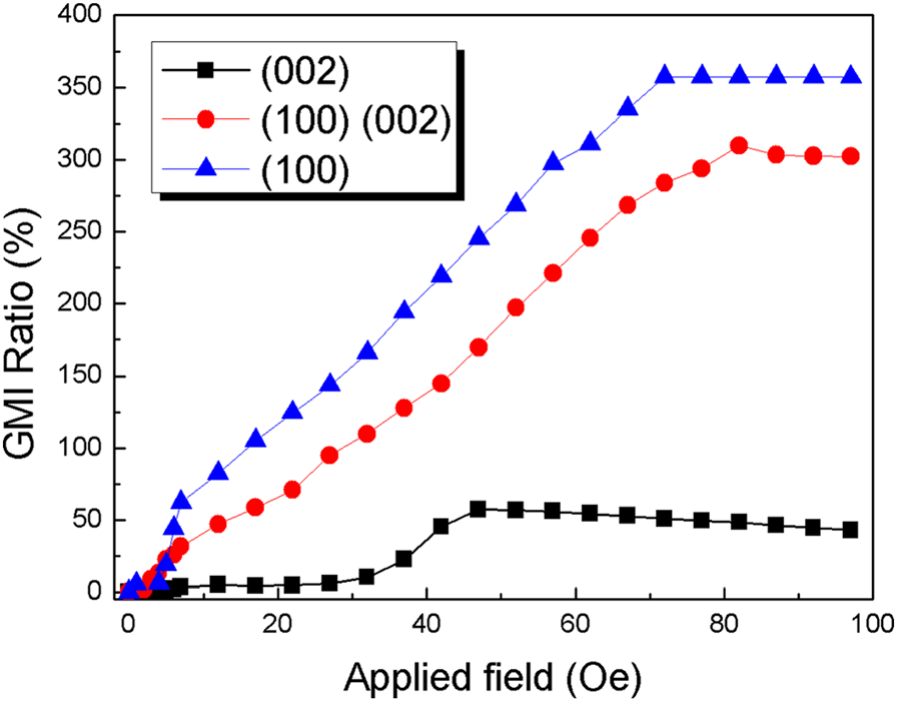
MI ratio of the nanobrush with 50-nm textured nanowires.

Figure 7 shows the magnetic field dependence of the MI ratio of the nanobrush fabricated by 50-nm textured Co nanowires and FeNi film. The 20-nm nanobrush shows the same characteristics, in which the best MI ratio appears in the nanobrush with (100)-textured nanowires. The maximum could reach more than 350% at a frequency of 10 MHz. The (002) texture shows the lowest MI ratio, which is only 52%, and the mixed structure shows a middle performance. Both the 20- and 50-nm nanobrushes show a similar tendency of MI curves: (100) and (002) textures can both enhance the MI ratio of the nanobrush, and the (100) texture shows the best results. MI property and magnetic field sensitivity strongly depend on the film's surface morphology and the combination of the nanowires and film. It may be the main reason that the sensitivity of the 50-nm nanobrush is not as good as that of other samples.

## Conclusions

The MI effect of the nanobrush with FeNi film and texture-controllable cobalt nanowires has been investigated. Cobalt nanowires with (100), (002), and mixed structures have been fabricated by different pH values and deposition temperatures. The optimized results of the (100)-textured nanobrush are 320% and 350% with 20- and 50-nm diameters, respectively. The phenomenon can be explained by the different distributions of transverse magnetic moments, induced by the exchange coupling effect between the interface of nanowires and film. Micromagnetic simulation shows the magnetic moment distribution when the nanowires act on the film. The parallel and perpendicular exchange coupling models are supposed to be the main reason of the different MI performances.

## Competing interests

The authors declare that they have no competing interests.

## Authors’ contributions

YZ, JD, and XJS did the study of the optimum conditions for nanobrush in the giant magnetoimpedance effect. YZ wrote the main part of the manuscript. QFL and JBW supervised the whole study. All authors discussed the results and implications and commented on the manuscript at all stages. All authors read and approved the final manuscript.

## Authors’ information

JBW and QFL are professors at the Institute of Applied Magnetics, Key Laboratory for Magnetism and Magnetic Materials of the Ministry of Education, Lanzhou University. YZ is a Ph.D. student.
